# Clinical study on localization of calcific tendinitis of the supraspinatus tendon based on three-dimensional reconstruction technology of CT scan

**DOI:** 10.3389/fsurg.2025.1534249

**Published:** 2025-08-12

**Authors:** Shicheng Jia, Xiaolei Liu, Menghao Li, Xin Mu, Jianjing Lin, Jiayou Chen, Shengbo Lu, Wei Li

**Affiliations:** ^1^ Department of Sports Medicine, Peking University Shenzhen Hospital, Shenzhen, Guangdong, China; ^2^ Shantou University Medical College, Shantou, China; ^3^ Department of Radiology, The First Affiliated Hospital of Shenzhen University, Health Science Center, Shenzhen Second People’s Hospital, Shenzhen, China; ^4^ Shenzhen University, Shenzhen, Guangdong, China

**Keywords:** calcific tendonitis, arthroscopy, 3D-CT scan, surgery, preoperative localization, shoulders

## Abstract

**Background:**

Arthroscopic resection of calcific lesions is an effective treatment for calcific tendonitis. However, accurately locating the calcific foci can be challenging. In this study, we propose the use of preoperative 3D-CT scan technology combined with intraoperative patient position and markings to precisely locate intraoperative lesions. The aim is to reduce exploration time and operative time.

**Methods:**

We retrospectively analyzed 51 cases of calcific tendonitis from inpatients at our department from June 2016 to January 2024. The patients were divided into a localization group (*n* = 26) and a non-localization group (*n* = 25) based on whether preoperative 3D-CT scan and intraoperative patient markings were used for lesion localization. In the localization group, the calcific lesions were located on the skin surface using the improved preoperative 3D-CT scan and intraoperative patient markings technique. In the non-localized group, a conventional surgical approach was performed. The intraoperative exploration time and operation time were compared between the two groups. ASES scores, VAS scores, and shoulder mobility were recorded before the operation, after the operation, and at the one-month postoperative follow-up.

**Results:**

The localization group had significantly shorter intraoperative exploration time and operation time compared to the non-localization group (*p* < 0.01). There were no significant differences in ASES scores, VAS scores, and shoulder mobility between the two groups (*p* > 0.05).

**Conclusion:**

The improved preoperative 3D-CT scan and intraoperative patient markings technique is a simple and effective method for localizing calcific tendonitis lesions before arthroscopic exploration. This technique can reduce exploration time and shorten operative time.

**Level of evidence:**

Level III; Cohort Study; Retrospective Study.

## Introduction

Calcific tendonitis is one of the most common causes of nontraumatic shoulder pain, primarily affecting women aged 30–60 years (1). It most commonly occurs in the supraspinatus tendon (2, 3), often triggered by minor trauma or overexertion. Calcific tendonitis causes severe shoulder pain and restricts joint movement. Approximately 2.7%–20% of patients with calcific tendonitis are asymptomatic, and 35%–45% are incidentally detected on simple radiographs (4), as symptoms arise following the formation of calcified deposits. When the calcific deposits exceed 1.5 cm in diameter, symptoms worsen and can lead to muscle spasms, tears, bursitis, and adhesive capsulitis. These complications significantly impact patients’ quality of life (5, 6). It is essential to differentiate the pain caused by calcific tendonitis from rare complications unrelated to disease progression, such as long head of the biceps pathologies or osteolysis of the greater tuberosity (7), as all of these conditions significantly affect patients’ quality of life. The conventional diagnostic approach involves using standard x-rays in the anterior-posterior, outlet, and axillary positions, as well as CT for follow-up (8, 9). MRI is utilized to identify possible complications, such as rotator cuff injuries (10). This approach aligns with clinical consensus, as it allows for lesion localization and assessment of deposition texture and morphology. Patients with acute exacerbation of the disease or failure of nonsurgical treatment, defined as persistent symptomatic calcific tendinitis after at least 6 months of nonsurgical treatment, including standardized nonsurgical treatment for at least 3 months, may require surgical intervention (11). Arthroscopic removal of calcified lesions provides significant pain relief and promotes postoperative shoulder function recovery (6). Therefore, arthroscopic treatment is a suitable option for cases where conservative treatment has failed and acute pain is unresponsive to conservative management.

However, arthroscopic treatment of calcific tendonitis still presents several challenges (12). The key issue lies in the rapid and accurate localization of the lesion during surgery to minimize operation time and iatrogenic injury (12). Currently, most surgeons rely on empirical localization, observing “storm” changes or using spinal puncture needles to locate “strawberry spots” and areas of calcific swelling (13). However, this traditional localization method heavily relies on experience and direct observation, which can lead to delays in the procedure and unnecessary trauma for inexperienced surgeons. As research and surgical techniques continue to advance, new preoperative localization methods have been proposed. Kayser concluded that preoperative ultrasound localization significantly improved treatment outcomes and increased the rate of successful removal of calcific foci (14). Ultrasound has been utilized to guide wire placement for preoperative localization of calcific foci (15). Similarly, Robert achieved significant results by using an internal ultrasound 10 mm probe for guidance during cleanup procedures (16). A recent study of ultrasound-guided arthroscopic resection of calcifying tendinitis showed successful results in terms of functional recovery and pain relief, but the effect of ultrasound-guided arthroscopic resection on exploration time and operation time was not explored (17). Imaging techniques are also widely employed in both diagnosis and surgical assistance. Although CT and x-ray imaging are commonly used for the diagnosis of calcific tendonitis, their true value in preoperative preparation for calcific debridement has not been fully appreciated (18). Moreover, due to changes in body position and the effects of necessary traction during surgery, the location of calcific foci that were directly marked and preoperatively localized often differs from the preoperative condition. The current trend in treatment involves targeted examination and localization for patients with different factors that may affect intraoperative localization, such as ligament and tendon strength and joint laxity. Considering that ultrasound and 3D-CT imaging are predominantly used in academia to assist with localization, exploring the further application of 3D imaging in localization and combining it with ergonomics to infer the location of calcific foci after postural changes may be a novel approach to consider.

Based on the findings from previous studies, we propose a method for locating calcific foci using preoperative 3D CT imaging and assessing the changes in position caused by postural adjustments during formal surgery. This approach involves utilizing geometric formulas to minimize operative time and reduce intraoperative injuries while ensuring the effectiveness of arthroscopic treatment.

## Materials and methods

The study was screened from inpatients with calcific tendonitis at our department from June 2016 to January 2024. We obtained 51 previous cases of calcific tendonitis and retrospectively analyzed the data. Cases were divided into localized (26) and non-localized (25) groups based on whether or not CT scans were used to localize the calcified foci preoperatively. The study conformed to the CONSORT (Consolidated Standards of Reporting Trials) statement. The study was approved by the Ethics Committee of Peking University Shenzhen Hospital (No. 2023-160). Eligible patients were aged between 18 and 65 years with a diagnosis of calcific tendonitis. In all patients, a suitable course of nonoperative management had failed, they should be assessed according to per inclusion or exclusion criteria (Table 1). Inclusion criteria:(1) Between 18 and 65 years of age; (2) Patients diagnosed with calcific tendonitis of the supraspinatus muscle based on clinical manifestations, medical history, physical examination and imaging examination; (3) After standardized conservative treatment, the patient still cannot get obvious relief or the symptoms are persistent; (4) Patients with calcified supraspinatus tendon during arthroscopy. Exclusion criteria: (1) The presence of severe osteoarthritis in the affected shoulder, history of previous shoulder surgery, cervical spondylosis, neurological diseases of the upper limb, and other disorders that affect the outcome; (2) Preoperative orthopantomogram or 3D CT scan of the shoulder joint cannot show the calcified foci of the rotator cuff, or cannot clearly show the position and size of the deposits through different planes; (3) Patients with arthroscopic findings of superficial calcification foci on the synovial margin or joint margin or obvious rotator cuff tear; (4) Patients with calcification foci larger than 1.5 cm in diameter; (5) Patients who are unable to tolerate surgery or have cognitive function or psychiatric disorders that prevent them from cooperating with follow-up visits. All surgery was performed by experienced, fellowship-trained shoulder surgeons.

**Table 1:** The general statistics collected within this study.

Variable		Localization group (n_1_ = 26)	Non-localization group (n_2_ = 25)	*P* value
Age $\left(\bar{x}\pm s\right)$ (y)		48.96 ± 9.54	45.60 ± 11.08	*P* > 0.05
Gender	Male	8 (31.00%)	10 (40.00%)	*P* > 0.05
	Female	18 (69.00%)	15 (60.00%)	*P* > 0.05
Duration of conservative treatment [Median(Q1,Q3)](m)		6.5 (6, 12)	6 (6, 9)	*P* > 0.05
ASES	Preoperative $\left(\bar{x}\pm s\right)$	52.24 ± 13.01	51.58 ± 13.04	*P* > 0.05
	Postoperative(1d) $\left(\bar{x}\pm s\right)$	86.36 ± 7.16	83.02 ± 7.10	
	1-month follow-up $\left(\bar{x}\pm s\right)$	91.1 ± 15.48	92.2 ± 3.61	
External rotation angle (°)	Preoperative $\left(\bar{x}\pm s\right)$	23.78 ± 5.71	25.76 ± 5.37	*P* > 0.05
	Postoperative(1d) $\left(\bar{x}\pm s\right)$	37.75 ± 3.49	38.74 ± 4.04	
	1-month follow-up $\left(\bar{x}\pm s\right)$	53.13 ± 4.29	54.06 ± 4.00	
Forward Flexion angle (°)	Preoperative $\left(\bar{x}\pm s\right)$	85.30 ± 6.64	84.4 ± 6.90	*P* > 0.05
	Postoperative(1d) $\left(\bar{x}\pm s\right)$	136.07 ± 7.16	136.9 ± 7.12	
	1-month follow-up $\left(\bar{x}\pm s\right)$	169.90 ± 5.68	170.69 ± 4.57	
VAS	Preoperative $\left(\bar{x}\pm s\right)$	8.08 ± 0.80	8.00 ± 0.58	*P* > 0.05
	Postoperative(1d) $\left(\bar{x}\pm s\right)$	2.12 ± 0.65	2.44 ± 0.65	
	Postoperative(1 m) $\left(\bar{x}\pm s\right)$	1.00 ± 0.52	1.00 ± 0.60	
Patients’ satisfaction $\left(\bar{x}\pm s\right)$		7.92 ± 0.85	7.88 ± 0.78	*P* > 0.05
exploration time $\left(\bar{x}\pm s\right)$ (s)		9.31 ± 2.94	336.96 ± 163.14	*P* < 0.05
Operative time $\left(\bar{x}\pm s\right)$ (min)		33.71 ± 5.09	37.95 ± 5.00	*P* < 0.05

### Localization

**Figure 1:**
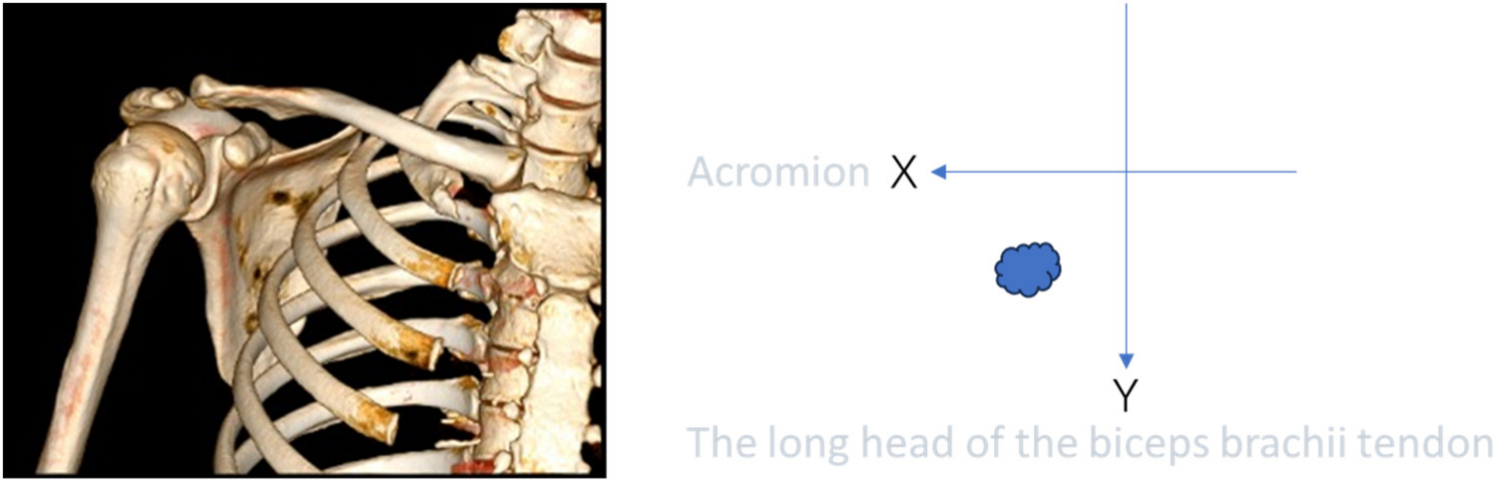
The *X*-axis represents the acromion, and the *Y*-axis represents the long head of the biceps brachii tendon. In this coordinate system, the position of calcifications is depicted by blue clouds. This is a simple schematic diagram.

**Figure 2:**
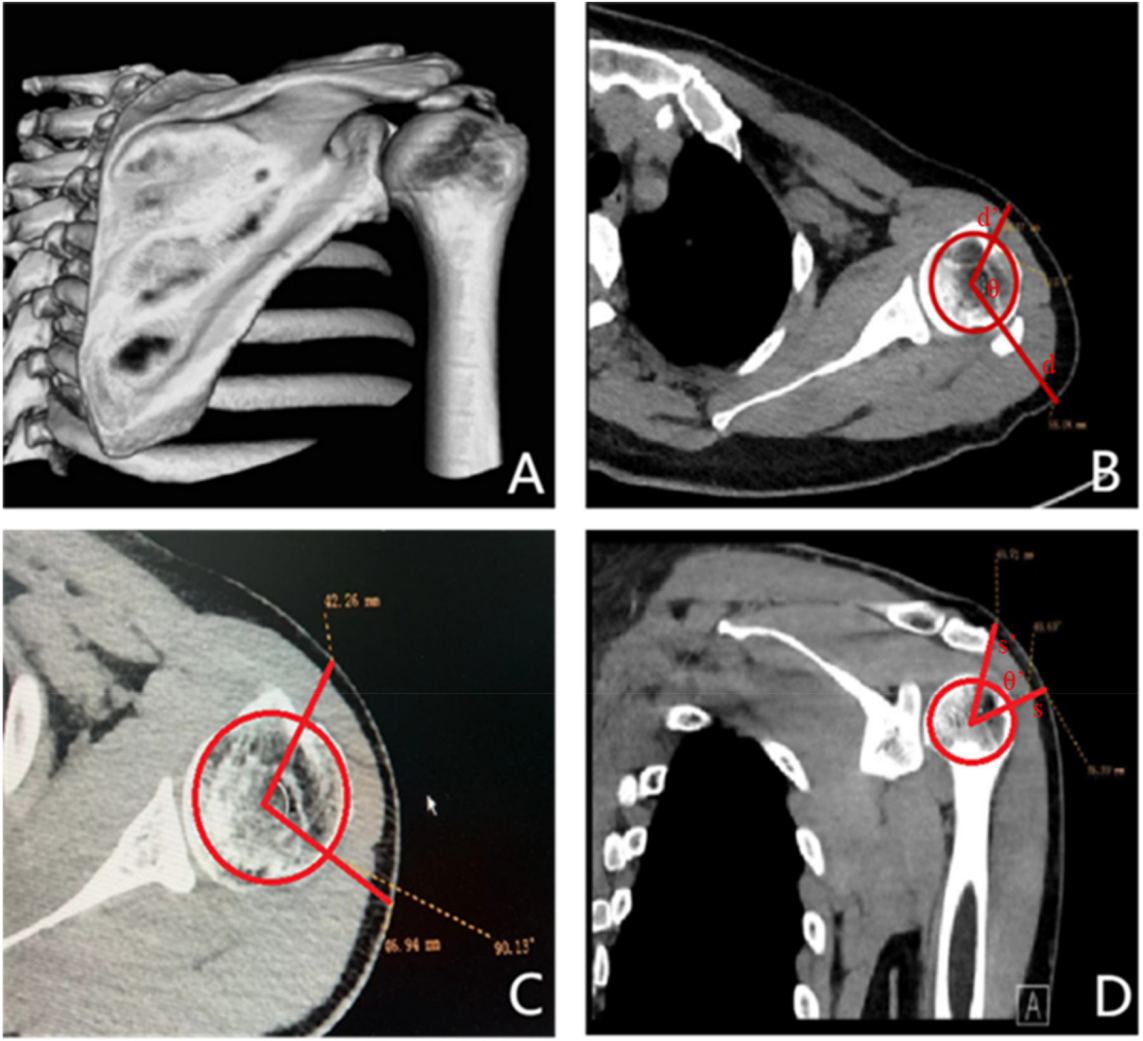
**(A)** This figure is a 3D CT image of the shoulder joint, showing calcium deposits within the shoulder joint. **(B,C)**: These figures show the cross-sectional CT of the shoulder joint and the measurements of d, d ‘and *θ*. **(D)**: This figure shows the coronal CT of the shoulder joint, measuring the value of s, s’, θ ’.

In the present study, we updated the preoperative localization method. The schematic diagram is presented in Figure 1. First, we performed preoperative 3D CT imaging (Figure 2A) of the patients’ shoulder joints, and judged the approximate location of the calcific foci based on the results of preoperative 3D CT. After that, Based primarily on the 3 projections of the CT scan, we measured the distance from the center of the humeral head to body surface through midpoint of the calcific foci(d) and interbicipital groove(d’), radius $R=\;(d+\;d^{\prime })/2$ in the preoperative CT image, and the angle between the two line segments was θ. The length of the calcific foci movement when rotate the shoulder could be approximated as the arc length $\;l=\;\pi R\cdot \frac{\theta }{180^{\circ }}$ (Figures 2B,C). And then we measured the distance from center of the humeral head to lateral border of the acromion (s’) and to body surface through midpoint of the calcific foci (s), raidus $R^{\prime }=(s+s{}^{\prime })/2$, and the angle between the two line segments was θ ’. The length of the calcific foci movement when rotate the shoulder could be approximated as arc $l^{\prime }=\pi R^{\prime }\cdot \frac{\theta }{180^{\circ }}$ (Figure 2D). In addition, we marked the position of the humeral head before and after pulling the upper limb, and measured the displacement distance of the humeral head. According to our experience, this distance typically ranged from 6 to 10 mm.

**Figure 3:**
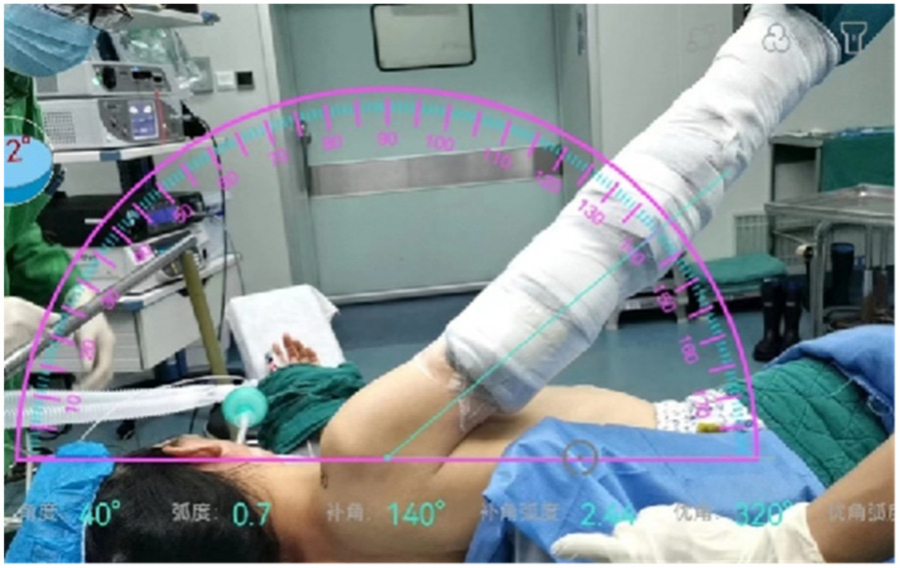
The patient was placed in the lateral decubitus with the affected arm abduction, and the abduction angle was measured by the software.

### Surgical methods

During routine arthroscopic surgery for calcific tendonitis, the patient is positioned in the lateral decubitus position. The affected shoulder is abducted at an angle measured by software and anteriorly flexed by 20°, with an average upper extremity traction of 3–5 kg (Figure 3). The surgical approaches used include the conventional posterior approach, anterior approach, and lateral approach.

**Figure 4:**
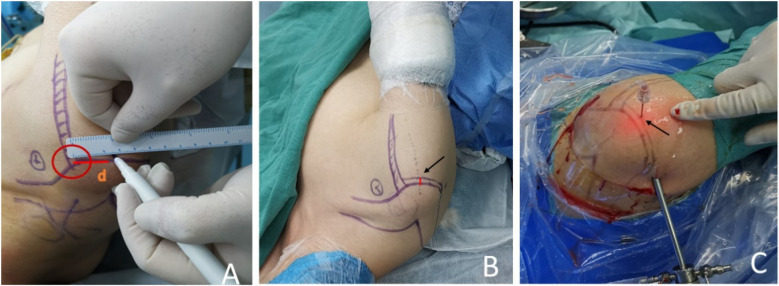
**(A)** The surgical approach was plotted preoperatively according to the precise positioning technology assisted by three-dimensional CT. **(B)** Adding the displacement distance of the humeral head caused by traction to the first position, the final position was determined. The arrow shows the distance where the humeral head moves down from the first position. **(C)** On the final position, we pierced the skin with the needle vertically. The arrow indicates the puncture point.

In the localization group, a surgical marker is used to mark the corresponding location on the patient's body surface. As the patient's arm rotates according to the requirements of shoulder arthroscopy and is visualized through CT scans, the initial position can be easily identified (Figure 4A). The final position is determined by adding the displacement distance of the humeral head caused by traction to the initial position (Figure 4B). These steps enable the determination of the vertical puncture position on the body surface and localization of the central position of the calcification lesion before surgery (Figure 4C).

**Figure 5:**
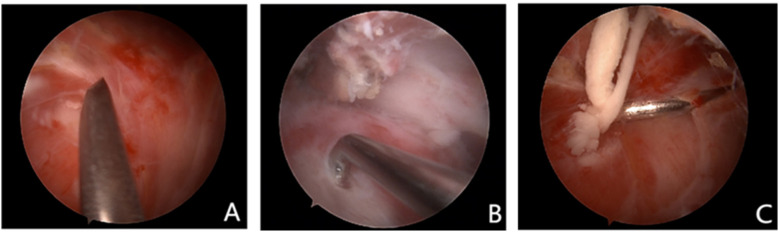
**(A)** This figure shows that the arthroscopy entered the subacromial space. **(B)** This figure shows that the calcified lesions were exposed under arthroscopy. **(C)** This figure shows that the calcified lesions were cleaned under arthroscopy.

In the non-localization group, conventional surgical treatment is performed according to established surgical guidelines. The glenohumeral joint is explored first, followed by entry into the subacromial space (Figure 5A). In the subacromial space, the subacromial bursa is cleaned, and then the calcified foci are exposed and removed (Figures 5B,C). If necessary, a C-arm x-ray machine may be used to confirm the cleanliness of the lesion.

### Postoperative rehabilitation and follow-up

Following the surgery, postoperative care included initiating progressive range of motion exercises and stretching. These measures aimed to facilitate the recovery and rehabilitation process. At the one-month follow-up appointment, several outcome measures were recorded to assess the effectiveness of the surgical intervention. The American Shoulder and Elbow Surgeons (ASES) score, which evaluates the functionality and pain levels of the shoulder joint, was assessed. Additionally, the abduction angle and forward flexion angle were measured to quantify the improvement in shoulder joint mobility and strength. These measurements provide objective data on the patient's progress and help evaluate the success of the surgical procedure.

### Standards

The primary outcomes of this study focused on the differences in exploration time and surgical time between the two groups. These metrics provide valuable insights into the efficiency and effectiveness of the surgical procedures performed. As for the secondary outcome measures, changes in the American Shoulder and Elbow Surgeons (ASES) scores were evaluated. These scores assess the functional improvement and pain levels in the shoulder joint, providing objective data on the postoperative outcomes. In addition, the angles of shoulder supination and external rotation were measured before the operation and at the end of hospitalization. These measurements offer quantitative information on the improvement in shoulder joint mobility and range of motion. To ensure the accuracy and eliminate potential observer bias, these outcome measures were assessed using objective measurement tools rather than relying on visual estimation.

Furthermore, subjective satisfaction assessments were conducted, with satisfaction scores ranging from 0 to 10. A score of 0 indicated an unsatisfactory outcome, scores ≤3 indicated mild satisfaction, scores ∼7 indicated moderate satisfaction, and scores ∼10 indicated high satisfaction. This subjective assessment provides insights into the patients’ perception of their surgical outcomes and overall satisfaction with the procedure.

By incorporating both objective and subjective outcome measures, this study provides a comprehensive evaluation of the surgical intervention's effectiveness, patient-reported outcomes, and satisfaction levels.

### Statistical analysis

A sample size calculation was performed using OSS sample data collected from patients previously undergoing arthroscopic calcific foci removement (*α* = 0.05, *β* = 0.80, sample size = 46). K-S test was used for normality test for all non-imaging data. Rank sum test was used for data that did not satisfy the normal distribution. For the data that met the normal distribution, we tested for homogeneity of variance. *T*-test was used for data that met the homogeneity of variance, and grouped designed test was used for the data that did not satisfy the homogeneity of variance. In addition, we used the difference of ASES, VAS and angle of shoulder joint movement as the standard to quantitatively calculate the improvement of the patient's symptoms after surgery (Table 1), conducting paired *t*-test or paired-rank sum test, according to the results of test for normal distribution. The final test results were enumerated and interpreted, and all the above statistical tests were performed in SPSS 26.0. *p* < 0.05 was considered statistically significant in each statistical analysis.

## Results

### General information

A total of 51 patients admitted for surgical treatment with the proposed diagnosis of “calcific tendonitis of the shoulder” or “calcification of the supraspinatus tendon” during June 2016 to January 2024 in this study. All patients received 6 months of conservative treatment without improvement, and the recurrent x-ray showed that the calcified foci did not subside, so they chose arthroscopic surgery. The details are demonstrated in Table 1.

### Statistical results

We used the Wilcoxon rank-sum test (Mann–Whitney *U* test) and found no significant differences between the two groups in terms of age, duration of conservative treatment, preoperative ASES scores, VAS scores, flexion, and external rotation angles (*P* > 0.05). Apart from significant differences in exploration time and surgery time (*P* < 0.05), the independent samples *T*-test did not show differences between the two groups in terms of ASES scores, VAS scores, flexion, and external rotation angles (*P* > 0.05). The paired *T*-test demonstrated significant differences in preoperative and postoperative ASES scores, VAS scores, flexion, and external rotation angles for both groups (*P* < 0.05), with significant improvement observed on the first day post-surgery. Patient satisfaction after surgery was rated as highly satisfactory.

## Discussion

Calcific tendonitis of the supraspinatus tendon is a prevalent condition, yet its exact pathogenesis remains elusive. The formation of calcific deposits primarily occurs within 1.0 cm proximal to the superior portion of the greater tuberosity of the humerus, specifically in the subacromial crest. This region is characterized by poor vascularity, making it particularly susceptible to stress-related damage. The combination of degeneration and cumulative strain further increases the likelihood of calcium deposition in this area.

Among the available therapies for calcific tendonitis, conservative treatments such as medications, corticosteroid injections, and physical therapy are typically attempted as initial options. Nonsteroidal anti-inflammatory drugs (NSAIDs) are commonly prescribed, although their long-term use carries risks of gastrointestinal, cardiovascular, and renal complications (19). While conservative treatment approaches have been widely used, they have not completely replaced surgical interventions. This is mainly due to the uncertainty surrounding their efficacy, as few high-quality clinical studies have demonstrated their superiority over surgery for symptomatic calcific tendonitis. With advancements in technology, extracorporeal shock wave therapy (ESWT) and Ultrasound-Guided Needling and Lavage (UGNL) have emerged as alternative treatment options. ESWT has been recommended as a second-line opion when conservative drug therapy fails, with reported success rates ranging from 60% to 80% in available studies (20, 21). However, the optimal pulse energy for ESWT remains inconclusive, and potential complications such as erythema, petechiae, and mild subdermal hematoma may occur. These complications often resolve on their own without medical intervention (22). UGNL is a technique that employs ultrasound guidance to remove calcium deposits from specific areas. It has demonstrated effectiveness in both short-term and long-term treatment of calcific tendinopathy (23). However, similar to ESWT, there is no established gold standard for ultrasound-guided percutaneous irrigation based on evidence-based medicine (6).

A recent clinical study indicated that both UGNL and ESWT were successful in improving function and reducing pain, with high satisfaction rates at 1-year follow-up. However, UGNL was found to be more effective in eliminating calcified deposits, while additional treatment was more common in the ESWT group (24, 25). Another study by Frassanito reported that combining Kinesio taping with ESWT appeared to enhance recovery from calcific tendinopathy of the rotator cuff and produced a faster treatment response compared to ESWT alone (26).

Overall, while conservative treatments are commonly employed as the initial approach for calcific tendonitis, the efficacy of these therapies compared to surgical interventions remains uncertain (27). Surgical treatment is comparable to non-surgical treatment for pain improvement, with the advantage being functional improvement (28). Ultrasound radiomics has been proposed to predict the success of US-guided percutaneous irrigation for calcific tendinopathy of the shoulder (29). Extracorporeal shock wave therapy and ultrasound-guided percutaneous irrigation have shown promise, but further research is needed to establish standardized guidelines and determine their optimal use.

In routine surgery for calcific tendinopathy, the use of shoulder radiography and CT is often necessary for diagnosis and preoperative localization. However, accurate localization of the lesion typically relies on the surgeon's intraoperative probing and empirical judgment. Currently, it is believed that arthroscopic visualization of an intra-tendon bulge with increased vascular tissue is a reliable marker of calcium deposition (30). Additionally, a “storm” effect observed in the interstitial space serves as an indicator of successful puncture of the calcified site (13). However, visual observation and empirical exploration carry certain risks, including the possibility of overlooking inconspicuous lesions under the microscope and potential injuries resulting from prolonged exploration time.

With the advancement of surgical techniques, several preoperative localization methods have been proposed, with ultrasound techniques being the most widely utilized. Kayser suggested that preoperative ultrasound localization significantly improves treatment outcomes and increases the clearance rate of calcification (14). However, their study did not demonstrate a positive effect of ultrasound localization on operative time and overall course reduction, possibly due to the learning curve associated with the technique. On the other hand, Sigg used ultrasound-guided wires for preoperative localization of calcified foci, which improved localization accuracy but introduced additional unpredictable risks (15). Robert (16) performed cleanup with a 10 mm internal ultrasound probe for guided localization, but the size and flexibility of the probe posed limitations. The 1 mm probe was not suitable, while the 10 mm probe was too large, although the proposed protocol was feasible in their case study. The modification of the probe by adding a sleeve could lead to damage during the exploration process, and current stage does not require probe modification for this procedure (31). Sabeti-Aschraf placed a puncture needle on the deposit under ultrasound guidance and marked it on the skin preoperatively using a ballpoint pen (32). This method is effective and reduces exposure to ionizing radiation from intraoperative radiography, but it carries a risk of greater variability, especially when deposits are located at the muscle-tendon junction, making them challenging to locate. In the pursuit of newer surgical methods, the academic community has predominantly focused on ultrasound techniques, which to some extent has limited the exploration of alternative approaches to improving surgical protocols. Ultrasound mainly provides two-dimensional images, making it difficult to fully understand the position and relationship of the lesion in three-dimensional space, which makes it difficult to meet the need for an in-depth understanding of the overall condition of the shoulder joint. The quality of its imaging is highly dependent on the operator's experience and skills, and there are difficulties in identifying deep calcified lesions; the clinical information provided is less comprehensive than that provided by CT in terms of ruling out other potential shoulder joint lesions, such as bone tumors, fractures, and so on. And compared to MRI, CT has the following advantages: better anatomical detail than MRI in terms of bone and calcification, easier to locate accurately, faster scanning speed, and higher accessibility and lower cost. In light of this, we propose an enhanced solution that involves utilizing preoperative 3D imaging to assist in localization.

In this study, a rigorous screening and random grouping process was implemented to effectively analyze the proposed surgical method while controlling variables. Although there were no significant differences in ASES, VAS, and patient satisfaction scores between the two groups regarding symptom improvement, the duration of surgery and intraoperative exploration time indicated that the method could effectively reduce operation time, particularly the time spent on intraoperative lesion exploration.

In conclusion, the pursuit of minimally invasive, individualized, and precise medical treatments has long been a goal embraced by clinicians in the field of surgery. In our study, we emphasized the importance of utilizing each patient's personalized imaging and 3D reconstruction results, along with a comprehensive understanding of the specific surgical traction force resulting from the displacement of the corresponding structures of the shoulder joint. This approach allowed us to accurately determine the surgical access and exploration location, thereby reducing the likelihood of intraoperative medical-induced injuries. Our study and method have effectively addressed the technical challenges associated with utilizing 3D imaging technology for the treatment of calcific tendonitis through shoulder arthroscopy, providing orthopedic surgeons with a reliable solution for effectively and precisely locating calcific foci, reducing operative time, minimizing intraoperative ionizing radiation exposure, and avoiding unnecessary harm. Consequently, this method can significantly reduce damage to the skin surface and internal structures caused by probe poking, ultimately leading to shorter operation times. This study has several limitations: it is retrospective, possesses a low level of evidence, and is conducted at a single center. The sample size is relatively small, and the investigation only evaluated short-term efficacy. The amount of radiation associated with CT cannot be ignored. Future research with extended follow-up is necessary to better understand the impact of 3D-CT localization on recurrence rates and long-term function. Additionally, other positioning modalities were not compared in this study, which presents an opportunity for future investigations to explore this and various rotator cuff component positioning techniques.

## Conclusion

The preoperative localization of calcified tendinitis lesions using the precise positioning concept, facilitated by a mathematical model supported by 3D-CT reconstruction technology, offers significant benefits in terms of reducing intraoperative lesion exploration time and overall operation time.

## Data Availability

The raw data supporting the conclusions of this article will be made available by the authors, without undue reservation.
